# Structural Features of Caspase-Activating Complexes

**DOI:** 10.3390/ijms13044807

**Published:** 2012-04-16

**Authors:** Hyun Ho Park

**Affiliations:** Department of Biochemistry, School of Biotechnology at Yeungnam University, Gyeongsan 712-749, Korea; E-Mail: hyunho@ynu.ac.kr; Tel.: +82-53-810-3045; Fax: +82-53-810-4769

**Keywords:** apoptosis, inflammation, caspase, apoptosome, DISC, PIDDosome, protein structure

## Abstract

Apoptosis, also called programmed cell death, is an orderly cellular suicide program that is critical for the development, immune regulation and homeostasis of a multi-cellular organism. Failure to control this process can lead to serious human diseases, including many types of cancer, neurodegenerative diseases, and autoimmununity. The process of apoptosis is mediated by the sequential activation of caspases, which are cysteine proteases. Initiator caspases, such as caspase-2, -8, -9, and -10, are activated by formation of caspase-activating complexes, which function as a platform to recruit caspases, providing proximity for self-activation. Well-known initiator caspase-activating complexes include (1) DISC (Death Inducing Signaling Complex), which activates caspases-8 and 10; (2) Apoptosome, which activates caspase-9; and (3) PIDDosome, which activates caspase-2. Because of the fundamental biological importance of capases, many structural and biochemical studies to understand the molecular basis of assembly mechanism of caspase-activating complexes have been performed. In this review, we summarize previous studies that have examined the structural and biochemical features of caspase-activating complexes. By analyzing the structural basis for the assembly mechanism of the caspase-activating complex, we hope to provide a comprehensive understanding of caspase activation by these important oligomeric complexes.

## 1. Introduction

Apoptosis, also called programmed cell death, is an orderly cellular suicide program that plays critical roles in embryonic development, immune regulation and cellular homeostasis [1–5]. Failure to control the apoptosis process, due to genetic mutations or abnormal expression of apoptosis-related proteins, can cause serious human diseases [2,6]. For example, hypo-activation of apoptosis is often associated with cancer, autoimmune disorders, and persistent viral infection, while hyper-activation of apoptosis is linked to many forms of neurodegenerative disorders such as Alzheimer’s disease and ischemic injury from stroke [7–12].

The process of apoptosis involves the sequential activation of particular proteases called caspases. For activation during the apoptotic process, caspases must form huge molecular complexes for self-activatation [13–18]. Two classical signaling pathways of apoptosis are well known: the extrinsic pathway and the intrinsic pathway [19]. The extrinsic pathway, which is also called the death-receptor dependent pathway, functions to remove unwanted or potentially dangerous cells. Death receptors are localized on cell surfaces and are activated by soluble or membrane attached death ligands [20]. Activated ligand-bound receptors form oligomeric caspase-activating complexes, called death-inducing signaling complex (DISC), which are formed by the receptor Fas, the intracellular adaptor FADD and caspase-8 or -10 [21,22]. In contrast, the intrinsic pathway, which is also known as the mitochodria-dependent pathway, is triggered by cellular damages or stress. It causes cytochrome c release from the mitochondria, which results in the formation of the caspase-9 activating complex apoptosome [23–25]. More recently, caspase-2 was shown to be involved in the intrinsic apoptosis pathway by acting upstream of the mitochondria [26]. Genotoxic stress is known as a trigger for the activation of capsase-2 [27]. Caspase-2 is activated by forming the caspase-activating complex, known as PIDDosome, which comprises PIDD, RAIDD and caspase-2 [27–29].

In this review, we summarized previous studies that examined the structural and biochemical features of caspase-activating complexes, including apoptosome, DISC, and PIDDosome. By analyzing the structural basis for the assembly mechanism of the caspase-activating complex, we hope to provide a comprehensive understanding of caspase activation by these important oligomeric complexes.

## 2. Caspase

**Figure 1:**
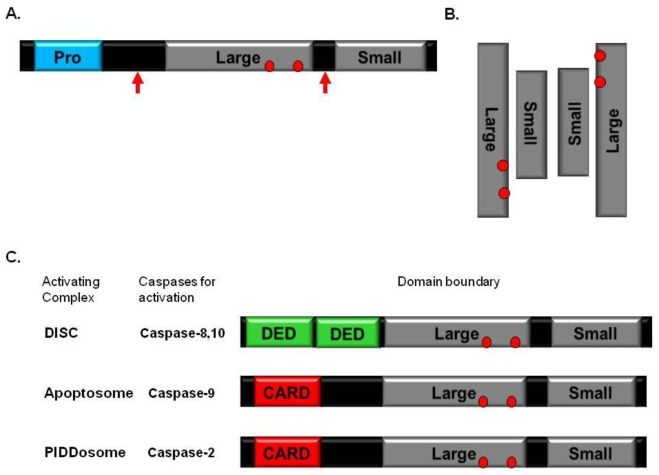
Initiator caspases and their activation. (**a**) Domain boundary of initiator caspase. Red arrows indicate proteolytic sites. Red circle indicates the catalytic sites; (**b**) Activated caspases. Red circles indicates the catalytic sites; (**c**) Domain boundary of initiator caspases and caspase-activating complex.

Apoptosis proceeds through characteristic morphological changes that are dependent on caspase activity. Caspases are cysteine proteases that specifically cleave proteins after aspartic acid residues [13,30]. They are synthesized as single-chain zymogens and require a highly regulated process for their activation (Figure 1A). Fully activated caspases are dimeric with two large subunits and two small subunits in general (Figure 1B). Both cleavage and dimerization are important to the integrity of the caspase active sites and therefore are required for caspase activation [31]. Cleavage and dimerization-dependent caspase activation is still a question under debate because several study showed that cleavage and dimerization of caspase-9 were not required for its activation [32,33].

Because caspases are the executioners of apoptosis, they are the key players in apoptotic cell death [34]. Caspases have been divided into two groups based on their sequence of activation and their roles in apoptosis; initiator caspases, such as caspase-2, 8, 9 and 10, and effector caspases, such as caspase-3 and 7. Initiator caspases, which have *N*-terminal prodomains for protein-protein interaction, are mostly monomeric in their pre-forms (Figure 1C). They are activated and auto-processed upon induced dimerization by recruitment to huge oligomeric signaling complexes known as DISC for caspase-8, 10 activation, Apoptososme for caspase-9 activation, and PIDDosome for caspase-2 activation [35] (Figure 1C).

## 3. Caspase-Activating Complexes and Death Domain Superfamily

The DD superfamily is one of the largest and most studied domain superfamilies [17,36]. It is currently comprised of four subfamilies, the death domain (DD) subfamily, the death effector domain (DED) subfamily, the caspase recruitment domain (CARD) subfamily and the pyrin domain (PYD) subfamily [36]. Based on a genome analysis, 32 DDs, 7 DEDs, 28 CARDs and 19 PYDs have been identified in the human genome [36]. The unifying feature of the DD superfamily is the six-helical bundle structural fold as first revealed by NMR structures of Fas DD [37], FADD DED [38], RAIDD CARD [39] and NALP1 PYD [40]. One of the main functions of the DD superfamily in apoptotic and inflammatory signaling pathways is the formation of oligomeric signaling complexes through homotypic interactions [18,19]. Caspase-8, 10 DED and Fas DD interact with FADD DED and FADD DD respectively for DISC assembly [41]. For the assembly of the apoptosome, caspase-9 CARD interacts with Apaf-1 CARD [42]. In the case of PIDDosome assembly, caspase-2 CARD and PIDD DD interact with RAIDD CARD and RAIDD DD, respectively [27].

## 4. DISC

**Figure 2:**
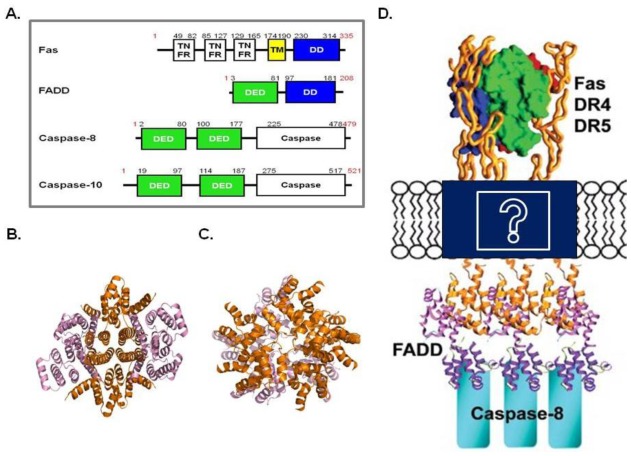
(**a**) Domain organization of the Death Inducing Signaling Complex (DISC) components, Fas, FADD, and caspase-8 or 10; (**b**) Overview of the Fas DD: FADD death domain (DD) complex structure solved under acidic pH conditions (Top view) (PDB id: 3EZQ). Four Fas DDs (orange color) are located inside of the complex structure and four FADD DDs (light pink color) are located outside of the complex structure; (**c**) Overview of the Fas DD: FADD DD complex structure solved under neutral pH conditions (Top view) (PDB id: 3OQ9). The top layer contains five Fas DD (orange color) and bottom layer contains five FADD DD (light pink color); (**d**) A model of the DISC for caspase-8 or 10 activation. The question mark was used to show the uncertainty in the stoichiometry and overall shape of DISC.

To remove healthy but unwanted or potentially dangerous cells, cell surface death receptors, which are members of the tumor necrosis factor (TNF) receptor superfamily, recognize death ligands in the beginning of the process. The interaction between death receptors and death ligands cause highly specific protein-protein associations to generate the oligomeric caspase-activating complex inside the cell, such as DISC. Death receptors, including Fas, contain a death domain (DD) in their intracellular regions, which is an adaptor module capable of homotypic interactions [43]. Upon ligand activation, the DD of Fas recruits the FADD adaptor protein via a homotypic interaction with the *C*-terminal DD of FADD (Figure 2A). FADD also contains an *N*-terminal DED that interacts with the tandem DED in the pro-domain of caspase-8 or -10 (Figure 2A). These interactions form the ternary DISC containing Fas, FADD and caspase-8 or -10 (Figure 2A). The recruitment and oligomerization of caspase-8 and -10 in the DISC result in its autocatalytic activation.

Two fundamentally different interaction modes between Fas and FADD have been detected recently. Based on the Fas DD:FADD DD complex structure, which was solved under acidic pH conditions, demonstrated the formation of DISC can only be regulated by Fas receptor clustering [44]. The structure was constructed with four Fas DD and four FADD DD (Figure 2B). Interestingly, in the formation of the complex, all of the contacts were mediated by Fas DD (Figure 2B). The key observation was that Fas DD underwent significant conformational changes when compared to the structure of the uncomplexed Fas DD. The structure determined in more recent studies were not in agreement with the Fas DD:FADD DD complex structure [45]. Unlike the previously elucidated structure, the new Fas DD:FADD DD complex was shown to form an asymmetric oligomeric structure composed of 5–7 Fas DD and 5 FADD DD, which suggests that the Fas/FADD structure solved under acidic conditions does not represent the physiological structure in solution (Figure 2C). The composition of DDs was similar to that of RAIDD DD:PIDD DD. Thus, the discrepancy between the current two Fas DD:FADD DD complex structures, where one was solved at neutal pH conditions and the other under acidic pH conditions, was likely due to the differences in the pH condition.

A more recent structural and biochemical study on DISC indicated that the dimer of the death ligand trimers may recruit six Fas DD and accommodate six FADD and six caspases for DISC assembly and caspase activation. However, the mysterious stoichiometry of the DISC-related proteins between the outside (trimeric death ligand and receptor interaction) and inside of the cell (4:4 or 5:5 assembly of Fas DD: FADD DD) and the structural discrepancy at different pH needs to be examined in more detail to better understand DISC assembly (Figure 2D).

## 5. Apoptosome

**Figure 3:**
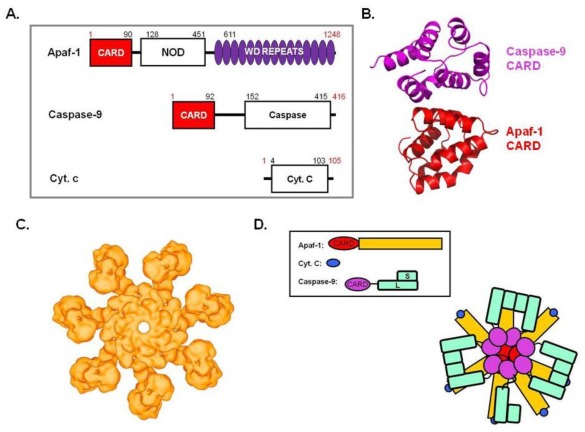
(**a**) Domain organization of the Apoptosome components, Apaf-1, Cyt. C, and caspase-9; (**b**) Overview of the caspase-9 caspase recruitment domain (CARD): Apaf-1 CARD complex structure (PDB id:3YGS). (**c**) Electron-microscopy structure of Apoptosome at a 12 Å resolution without caspase-9. The apoptosome adopts a wheel-like structure; (**d**) A model of the Apoptosome for caspase-9 activation. Schematic of caspase-9 dimerization to show proximity-induced dimerization in the Apoptosome.

The mitochondria are the central players in the intrinsic apoptotsis pathway and caspase-9 is major initiator caspase in this process. Leakage of resident proteins from the mitochondrial intermembrane space to the cytosol, which can occur for many reasons, such as irreversible DNA damage, survival factor withdrawal, hypoxia, and UV irradiation, is the one of the main initial events for intrinsic apoptosis. One of the most important mitochondrial proteins that get released during intrinsic apoptosis is cytochrome C, which is a bifunctional protein that functions in electron transport and apoptosis. The release of cytochrome C to the cytosol helps to assemble the Apoptosome, which contains Apaf-1, caspase-9, and cytochrome C [46] (Figure 3A). Apaf-1, the central component of the apoptosome, contains an *N*-terminal CARD, an expanded nucleotide-binding domain and a *C*-terminal WD40 domain (Figure 3A). Apaf-1 CARD is responsible for the interaction with the caspasae-9 CARD, which is essential to the recruitment and the activation of caspase-9 (Figure 3B). A structural study on the Apaf-1 CARD: caspase-9 CARD complex revealed that they form a hetero-dimeric interaction, which is unlike DD complex in DISC complex (Figure 3B) [47]. The most recent study, however, showed the possibility for the formation of super-helical complex between Apaf-1 CARD and caspase-9 CARD [33]. The nucleotide-binding domain of Apaf-1 has been shown to be responsible for oligomerization in the presence of cytochrome C and dATP. In the absence of signal, Apaf-1 exists as a monomer. However, during the apoptotic process, after the release of cytochrome C from mitochondria, Apaf-1 oligomerizes to form a heptameric ring-shaped molecular platform, called an Apoptosome, which contains seven Apaf-1, each bound to one molecule of cytochrome C (Figure 3C and 3D) [24,48]. Recent structural studies showed that the assembly of apoptosome itself (without caspase-9) does not require Apaf-1 CARD: caspase-9 CARD interaction, nor does it require Apaf-1 CARD: Apaf-1 CARD interaction (Figure 3D) [33,49,50]. The stoichiometry of caspase-9 in apoptosome is unclear. The caspase-9 to Apaf-1 ratio could be from 5:7 to 7:7 [33].

## 6. PIDDosome

**Figure 4:**
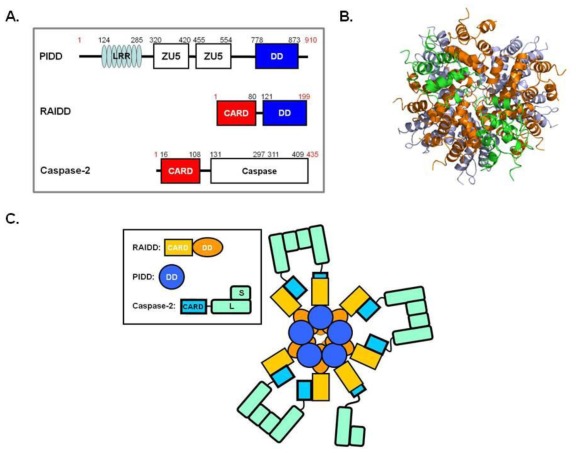
(**a**) Domain organization of the PIDD, RAIDD, caspase-2, which is the PIDDosome component; (**b**) Overview of the RAIDD DD: PIDD DD complex structure (Top view). The top layer contains two RAIDD DD (green color), the middle layer contains five RAIDD DD (orange color), and bottom layer contains five PIDD DD (light blue color) (PDB id: 2OF5); (**c**) A model of the PIDDosome for caspase-2 activation. Caspase-2 are schematically dimerized to show proximity-induced dimerization in the PIDDosome.

Caspase-2 is an initiator caspase and the most evolutionarily conserved caspase [26]. Caspase-2 acts upstream of the mitochondria and is involved in Bid cleavage and Bax translocation, which results in cytochrome c release during genotoxic stress [51]. The caspase-2 activating complex, PIDDosome, is composed of three protein components, PIDD, RAIDD and caspase-2 (Figure 4A). It is assembled via a DD: DD interaction between RAIDD and PIDD and a CARD: CARD interaction between RAIDD and caspase-2 (Figure 4A). PIDD DD is not only essential for the activation of caspase-2, but can also interact with the DD of RIP1, a kinase implicated in the activation of NF-κB [52]. PIDD appears to act as a molecular switch, controlling the balance between life and death upon DNA damage. Full length PIDD contains 910 residues with seven leucine rich repeats (LRRs), two ZU-5 domains and a *C*-terminal DD. It is often auto-processed via an intein-like mechanism into shorter fragments of 51 kD, 48 kD and 37 kD [53]. Auto-cleavage of PIDD determines the outcome of the downstream signaling events. PIDD-CC containing the *C*-terminal DD is sufficient for PIDDosome formation and caspase-2 activation [54].

The crystal structure of the PIDDosome core, which is composed of seven RAIDD DDs and five PIDD DDs, has been determined [18] (Figure 4B). The DD complex is divided into three layers; five PIDD DDs at the bottom, five RAIDD DDs in the middle, and two additional RAIDD DDs at the top (Figure 4A). Interestingly, this DD complex does not possess a distinct fivefold symmetry. Instead, the complex consists of two different types of unique rotation angles, one rotating around 84° and translating down the axis and the other rotating around 54° and translating up the axis. The complex is formed by three different types of interactions that are classified based on the regions involved in the interaction.

Based on the current structural and biochemical information on PIDDosome, it might be possible that five or seven RAIDD interact with five PIDD DD and five or seven caspase-2, forming the round shape caspase-2 docking station (Figure 4C). The molecular structure of the ternary complex, containing RAIDD, PIDD, caspase-2, should be solved to provide clear evidence of the stoichiometry, shape of the PIDDosome, and activation mechanism of initiator caspases.

## 7. Conclusions

The apoptosis process is mediated by sequential activation of caspases. To understand apoptotic cell death, therefore, many studies have examined the caspase activation mechanism. Despite intense structural and biochemical investigations, there are still many unknown features of the caspase and caspase activation mechanism. This review summarizes the most recent structural studies on the caspase-activating complexes. The key common event for activating initiator caspases is oligomerization, which allows the formation of activating dimmers. The recruitment of caspase by those caspase-activating complexes results in a local increase in the concentration of caspase, which subsequently become activated by proximity-induced dimerization.

The assembly mechanism of DISC, Apoptosome, and PIDDosome has been examined in several prevoius structural studies. For DISC assembly, it is possible that a dimer of the death ligand trimers binds to six Fas DD and accommodates six FADD and six caspases, resulting in caspase activation. However, because of the structural changes in the DISC under different pH conditions, the process of DISC formation is still unclear. The apoptosome, which is a heptameric ring-shaped molecular platform, contains seven Apaf-1, each bound to one molecule of cytochrome C and one caspase-9. PIDDosome is composed of five or seven RAIDD, five PIDD DD, and five or seven caspase-2. However, the structure of the ternary complex, containing RAIDD, PIDD, caspase-2, still needs to be determined.

Beside apoptotic caspases, there are caspases involved in inflammation, which are called inflammatory caspases, including caspase-1 and -5. Similar to the activation process of apoptotic caspases, activation of inflammatory caspases occurs throught the formation of huge caspase-activating complexes, called inflammasomes. One of the most well studied inflammasome is the NALP3 inflammasome, which is composed of three components, NALP3, ASC, and caspase-1 [55]. NALP3 is expected to function as intracellular danger signal sensor and consists of an *N*-terminal PYD domain, an intermediate NATCH domain, and *C*-terminal leucine-rich repeats. ASC is a bipartite adaptor protein that consists of an *N*-terminal PYD domain and a *C*-terminal CARD domain. Caspase-1 contains a CARD domain at the *N*-terminus. Although it has been known that NALP3 inflammasome is assembled via a PYD:PYD interaction between ASC and NALP3 and a CARD:CARD interaction between ASC and caspase-1, no structural information of the inflammasome complex is available. It might be very interesting to determine if the structure of the inflammasome is similar to those of DISC, Apoptosome, and PIDDosome. Recently introduced protein-protein database for the death domain superfamily will be helpful to study the death domain superfamily mediated formation of caspase-activating complexes [56].
